# Irritant Contact Dermatitis Risk of Common Topical Traditional Chinese Medicines Used for Skin-Lightening: A Pilot Clinical Trial with 30 Volunteers

**DOI:** 10.1155/2014/609064

**Published:** 2014-04-10

**Authors:** Kao-Sung Tsai, Tzu-Chun Lin, Meng-Tse Wu, Jui-Lung Shen, Ming-Ya Mao, Huey-Yi Chen, Yung-Hsiang Chen, Wen-Chi Chen

**Affiliations:** ^1^School of Chinese Medicine, Graduate Institute of Chinese Medicine, Graduate Institute of Integrated Medicine, College of Chinese Medicine, China Medical University, Taichung 40402, Taiwan; ^2^Departments of Dermatology, Medical Research, Obstetrics and Gynecology, and Urology, China Medical University Hospital, Taichung 40402, Taiwan; ^3^Department of Dermatology, Taichung Veteran General Hospital, Taichung 40705, Taiwan

## Abstract

Topical traditional Chinese medicine- (TTCM-) related contact dermatitis is not uncommon but ignored. Patch and photopatch tests using 6 individual herbal ingredients and Bai-Zhi-Kao (BZK; **白** **芷** **膏**), a skin-lightening TTCM preparation, were conducted on 30 participants. Twenty-five subjects showed at least 1 positive reaction, including 6 (20.0%) participants who reacted to BZK. The majority reacted to Radix *Ampelopsis japonica* (Bai-Lian; **白** **蘞**) (60.0%), whereas few reacted to Rhizoma Bletilla striata (Bai-Ji; **白** **芨**) (16.7%), Rhizoma *Atractylodis macrocephalae* (Bai-Zhu; **白** **朮**) (10.0%), Radix *Angelicae dahuricae* (Bai-Zhi; **白** **芷**) (3.3%), and Herba asari (Xi-Xin; **細** **辛**) (3.3%). In the photopatch test, 3 participants (10.0%) reacted positively to BZK and 10 to ≥1 constituent; however, all reacted to Radix *Angelicae dahuricae* (26.7%), Radix *Ampelopsis japonica* (13.3%), and Rhizoma Bletilla striata (3.3%). In contrast, no subjects showed positive reactions to Sclerotium Poria cocos (Bai-Fu-Ling; **白** **茯** **苓**). Thus, BZK and its constituents might present potential latent risk of contact dermatitis owing to the possible presence of Radix *Ampelopsis japonica* and Radix *Angelicae dahuricae*. Furthermore, TTCMs, particularly cosmetic products, must be used carefully, with ample warning of potential contact dermatitis risk.

## 1. Introduction

During the last few decades, cosmetics derived from or partially composed of topical traditional Chinese medicines (TTCMs) have been widely used in skin care, despite the lack of parallel human clinical trials [1–3]. However, research has revealed that allergic dermatitis and irritant contact dermatitis are the most common adverse events associated with TTCMs [4, 5]. Many variants of traditional Chinese medical formulas contain complex combinations of individual ingredients from multiple herbal plant constituents (in both crude and galenic extracted forms), thus limiting the scope of clinical investigation [6]. In addition, studies on TTCMs and other herbal medicine allergies are also problematic because of the limited number of commercially available standardised patch test substances and the danger of active sensitisation when testing with botanical medicines, their constituent parts or individual extracts.

Although TTCMs are regarded as critical allergens by dermatologists, the number of affected cases reported is relatively small. Furthermore, safety, adverse effects, standards, and quality control are issues of concern, and the evaluation of all of these factors should be mandatory in herbal medicine. For example, Bai-Zhi-Kao (BZK; *白芷膏*), a currently commonly used TTCM for skin lightening in Chinese communities, including those in Taiwan and China, had been mentioned in an ancient official Chinese pharmacopoeia (Sheng-Ji-Zong-Lu; *聖濟總錄*, A.D. 1,117). This topical Chinese medicinal formula is composed of 7 crude botanical components, including Radix* Angelicae dahuricae* (Bai-Zhi; *白芷*), Rhizoma* Atractylodis macrocephalae* (Bai-Zhu; *白朮*), Sclerotium* Poria cocos* (Bai-Fu-Ling; *白茯苓*), Rhizoma* Bletilla striata *(Bai-Ji; *白芨*), Radix* Ampelopsis japonica* (Bai-Lian; *白蘞*),* Herba asari* (Xi-Xin; *細辛*), and Rhizoma* Typhonium giganteum* (Bai-Fu-Zi; *白附子*). In the ancient pharmacopoeia, this mixture had been used for a relatively long period (used as a mask at night and rinsed off the next morning). The herbal constituents of this topical Chinese medicinal formula are known to cause appreciable antityrosinase activity and suppress tyrosinase synthesis [7–10]; thus, many BZK-based pharmaceutical and cosmetic products such as BZK medicinal powder and extracts mixed with aqueous, petrolatum or olive-oil vehicles are prescribed by traditional Chinese medicine practitioners in the Chinese community.

Herbal medicine is a widely used modality of complementary and alternative medicine. Along with the rapid growth of consumption, the safety of herbal medicines has become a highlighted topic. Thus, by employing patch and photopatch tests on healthy volunteers, this pilot clinical study was designed to evaluate the potential incidence of contact dermatitis resulting from some TTCMs commonly used.

## 2. Patients and Methods

### 2.1. Study Design and Subjects Selection

This pilot clinical study was conducted from April to May 2008, and volunteers were recruited from the outpatient clinic of the Dermatology Department of China Medical University Hospital in Taiwan. The inclusion criteria were age of 20–65 years and absence of any illnesses. The exclusion criteria were topical or systemic use of corticosteroids or antihistamines within 4 weeks of the start of the study, intake of immunosuppressive drugs, intense ultraviolet exposure on the back, prior eczematous dermatitis, actinic dermatitis or severe sunburn injury, and infectious disease at the patch test site on the back. In addition, pregnant and lactating women were also excluded. No patients were allowed to take corticosteroids or antihistamines during the study period. The Institutional Review Board (IRB) of the hospital approved the study (DMR96-IRB-186), and the subjects provided signed informed consent.

### 2.2. Herbal Materials and Preparation of Reagents

In the present study, the tested BZK was composed of 6 individual botanical components, including Radix* Angelicae dahuricae*, Rhizoma* Atractylodis macrocephalae*, Sclerotium* Poria cocos*, Rhizoma* Bletilla striata*, Radix* Ampelopsis japonica,* and* Herba asari*, which were further examined individually. Because of the potential preclinical toxic effects of Rhizoma* Typhonium giganteum* [11, 12], this component was excluded from the study, according to the IRB restriction.

It must be noted that only a few references to the patch test concentration of “cosmetic” TTCM substances are available in the literature. Patch test is used in traditional medicine to confirm the cause of contact dermatitis in “as-is” preparations and diluted substances [13]. Many substances used in everyday life, such as cosmetics and medications, are conventionally used without dilution [14]. In the present study, BZK and its constituents were subjected to patch test at an undiluted concentration in a 50% petrolatum mix to mimic their traditional daily use, according to the ancient pharmacopoeia.

The authenticated (appearance, microscopic characteristics, and thin-layer chromatography) samples of the individual herbal constituents, complying with specifications given in the Chinese pharmacopoeia, were provided by Sheng Foong Pharmaceutical Co., Ltd (Proof certification according to ISO17025/TAF, Ilan County, Taiwan). Briefly, the 6 dried herbal constituents (450 g for each constituent) were ground (5 min) into powder of 80 meshes by using herb-grinding machines (IKA A11 grinder; IKA, Germany). Each of the ground substances was homogeneously mixed with the warm (60°C) petrolatum base at a 1 : 1 ratio by weight. Likewise, the BZK formula was prepared in a similar manner from 6 equivalent 50 g herbal constituents and a 300 g petrolatum base. All the petrolatum-based reagents were prepared by Sinphar Pharmaceutical Co., Ltd. (Ilan County, Taiwan) in accordance with Good Laboratory Practice and stored in a refrigerator below 4°C until use. As a negative control, a sample of petrolatum base was employed. All the reagents were labelled as per their registration number.

### 2.3. Patch and Photo Patch Testing Procedures

**Figure 1:**
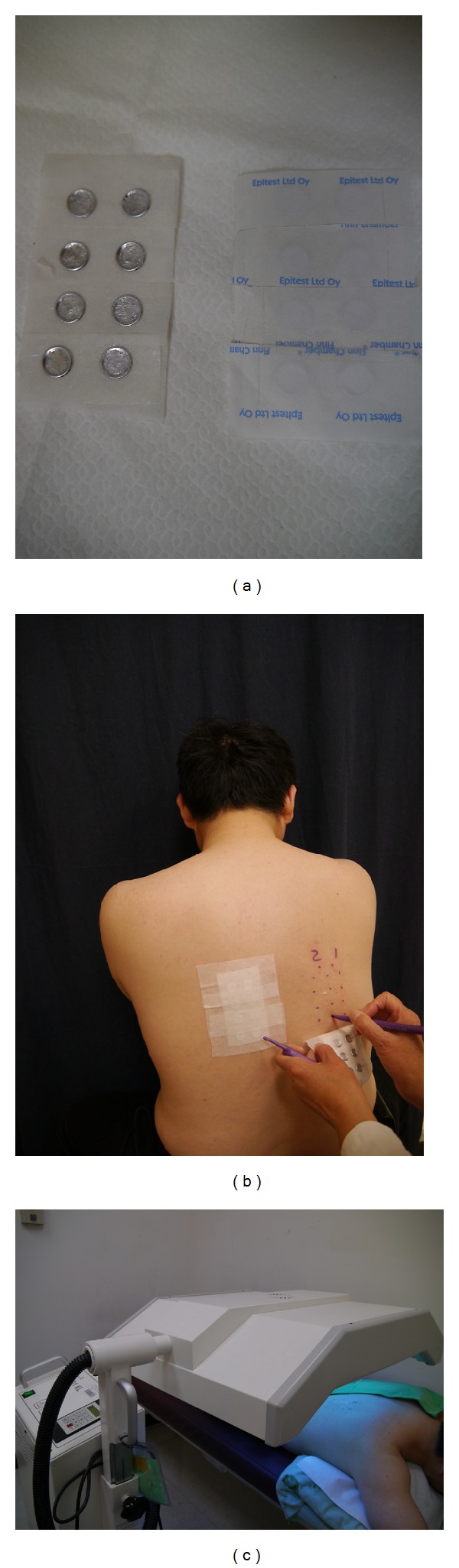
Patch and photopatch testing on an unaffected area of the upper back. (a) Each reagent placed within 8 mm Finn Chambers on Scanpor tape was then fixed with Scanpor tape and secured with 3M tape. (b) Each reagent was applied in duplicate to the left and right side of the upper back of each subject. (c) Photopatch tests were applied on the right side of the backs of subjects who were irradiated with 5 J/cm^2^ UVA on Day 1.

The subjects underwent patch and photopatch test on an unaffected area of the upper back. For patch tests, 50 mg of each reagent was placed within 8 mm Finn Chambers on Scanpor tape (Epitest Ltd., Tuusula, Finland), which were then fixed with Scanpor tape and secured with 3M tape (Figure 1(a)). Each reagent was applied in duplicate on the left and right side of the upper back of each subject (Figure 1(b)) for 24 h. The patches were then uniformly removed from side-to-side. Photopatch tests were applied on the right side of the backs of the subjects who were irradiated with 5 J/cm^2^ UVA (Waldmann UV801KL; Villing-Schwenningen, Germany) on Day 1 (Figure 1(c)).

### 2.4. Assessment

**Figure 2:**
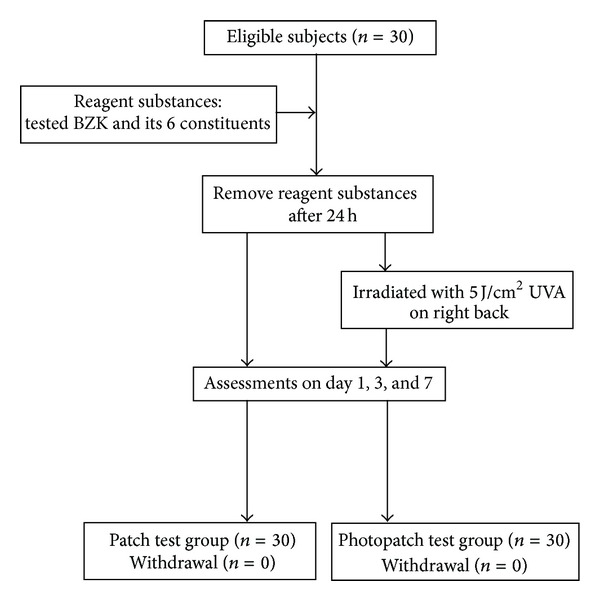
The flow chart of this study.

The patches were removed on Day 1 and readings were taken on Days 1, 3, and 7 by 2 experienced dermatologists. The test reactions were graded according to the guidelines of the International Contact Dermatitis Research Group [15]. Throughout the study period, all the tested cutaneous conditions and adverse effects were noted and recorded by using a digital camera. The flowchart describing the protocol is given in Figure 2.

### 2.5. Statistical Analyses

The study participants were characterised by using descriptive statistics (mean ± standard deviation). The chi-square test and Fisher exact test were used to compare the reactions to BZK and its constituents. A phi coefficient was determined to estimate the relationship between the reactive patch tests produced by the formula, BZK, and its constituents. Statistical significance was indicated by *P* < 0.05. All statistical analyses were carried out by using the software SPSS Statistics 17.0 (SPSS, Chicago, IL, USA).

## 3. Results

**Figure 3:**
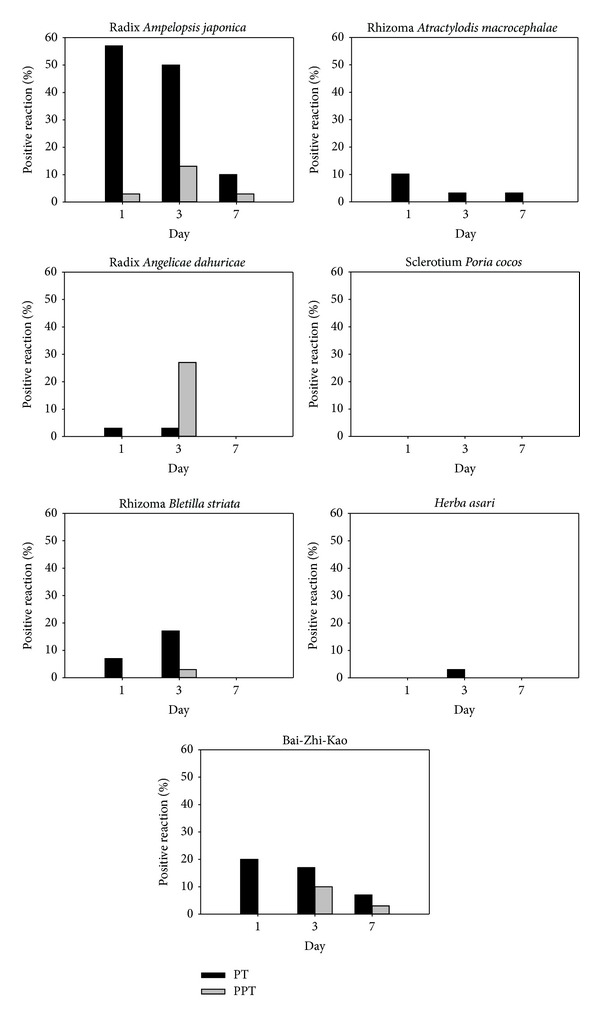
The positive reaction rate in all assessment days (Day 1, 3, and 7).

The average age of the participants was 35.2 ± 10.7 years (range, 21–64 years), with the majority being women (*n* = 25; 83.3%), and there were no dropouts. The detailed data obtained on all assessment days (Days 1, 3, and 7) are given in Figure 3, and the results of the patch and photopatch tests are summarised in Table 1.

**Figure 4:**
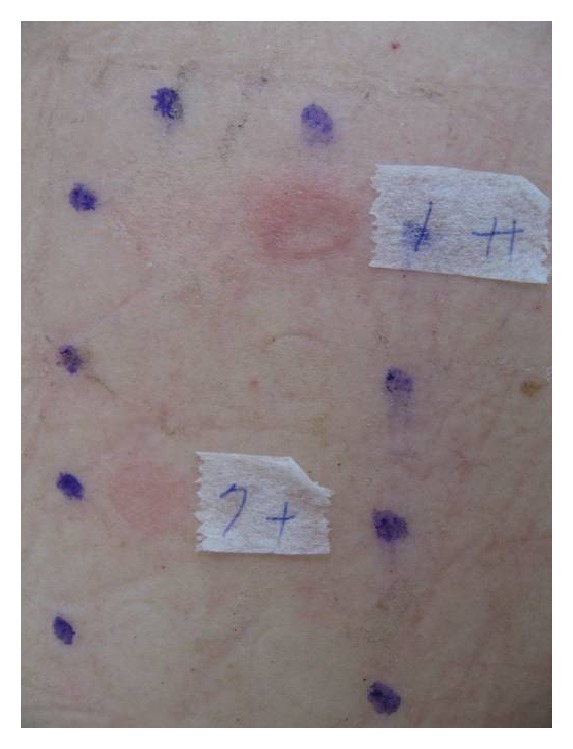
Patch test reactions in patient number 17 showed strong positive reaction (++) to the label number 1 substance and positive (+) to label number 7 substance.

Of the 30 subjects, 25 (5 men and 20 women) exhibited at least 1 positive reaction to the tested patches. Among them, 6 (20.0%) showed reactions to BZK. To identify the differences between the reactions to different BZK constituents, the individual reactions of the herbal constituents of this formula were analysed. The majority showed positive reactions to Radix* Ampelopsis japonica* (*n* = 18; 60.0%), followed by Rhizoma* Bletilla striata* (*n* = 5; 16.7%), Rhizoma* Atractylodis macrocephalae* (*n* = 3, 10.0%), Radix* Angelicae dahuricae* (*n* = 1; 3.3%), and* Herba asari* (*n* = 1; 3.3%). Furthermore, there were strong positive reactions (++) in 4 subjects, all of whom reacted to Radix* Ampelopsis japonica* (Figure 4). The positive patch test results between BZK and Radix* Ampelopsis japonica* were almost significant (*P* = 0.07; Fisher's exact test). The value of the phi coefficient was 0.408, indicating a moderate association of positive patch tests between BZK and Radix* Ampelopsis japonica*. In contrast, Sclerotium* Poria cocos* did not show any positive reaction in these subjects. All the control patch results with the petrolatum base were negative.

All the 9 subjects with positive reactions to the BZK patch or photopatch test also reacted to at least 1 of the constituents, including 4 subjects who were reactive to at least 2 constituents in the patch test. In contrast, the patch test did not reveal any positive reaction to the second-ranked reactive constituent Rhizoma* Bletilla striata*. Similarly, with regard to Radix* Angelicae dahuricae* and* Herba asari*, the number of reactive subjects was very small (for both,* n* = 1).

In the photopatch test series, 3 subjects (10.0%) showed positive reactions to BZK, 10 (33.3%) showed positive reactions to at least 1 constituent, and all showed positive reaction to Radix* Angelicae dahuricae* (*n* = 8; 26.7%), Radix* Ampelopsis japonicae* (*n* = 4; 13.3%), and Rhizoma* Bletilla striata* (*n* = 1; 3.3%). On the other hand, no positive reactions to the other 3 constituents were noted.

## 4. Discussion

The adverse effects caused by herbal medicines constitute an important, yet neglected, issue that deserves further investigation. Till date, a thorough method for the assessment of individual Chinese herb constituents, such as a patch test screening series for identifying certain contact sensitivities, has not yet been reported. Investigators often encounter problems when patch-testing TTCMs, because it is difficult to determine the correct reagent concentrations and vehicles. In the present study, the BZK constituent that caused maximum reactions was Radix* Ampelopsis japonica,* with approximately 60% subjects showing positive reactions to this compound. Among the patch-tested positive subjects, strong positive reactions (more than ++) to Radix* Ampelopsis japonica* were more frequent than those to the other constituents. This indicated that Radix* Ampelopsis japonica* has the greatest potential among all the constituents of BZK to induce contact sensitivities.

The natural compositions of herbal formula are comminuted, powdered, or galenic extracts of the whole or specific parts of a plant [16]. Recently, the potential use of TTCMs in developing new skin-care cosmetics has been emphasised [17]. The fact that women are exposed to more cosmetics and toiletries [18] explains their predominance among the subjects of the present study. Nonetheless, patch test results showed that 76.7% of our subjects were reactive to the formula or to its individual herbal constituents. This finding contradicts the common misconception that TTCM is harmless because of its natural composition and minimal side effects.

Further improvements in the safety of TTCM products can be achieved by replacing or removing the constituents that are associated with a higher risk of contact dermatitis, thus simplifying the composition of TTCM by decreasing the concentration of those constituents or by stating the potential adverse effects of TTCM products on their packages [13]. The fact that contact dermatitis resulting from the use of TTCMs is associated with a particularly high rate of patch test-positive reactions to individual constituents suggests that patients with positive patch test reactions to TTCM constituents or formulas should be advised to avoid or be more cautious when using them.

The BZK reagent tested in our study was composed of 6 individual/single botanical components with proven sun-protective qualities (data not shown), and as many as 40.0% subjects who underwent photopatch test showed reactions to the formula or its individual constituents. Therefore, we believe that these herbal remedies might have the potential to enhance photosensitisation [19]. In the photopatch test of individual constituents, a positive reaction was observed in 33.3% subjects. Among the constituents examined, Radix* Angelicae dahuricae* is known to contain furocoumarins such as imperatorin, isoimperatorin, and alloimperatorin, which are potentially strong photocontact sensitizers [20–22] that also confer photoallergenic capability. This individual constituent exhibited the highest positive rate in the photopatch test. It is known that the action spectrum of contact photodermatitis is related to the level of exposure to ultraviolet radiation and the concentration of harmful agents [23]. Hence, the adverse effect of photosensitivity should be more carefully considered when defining TTCM application.

The present study was observed to have several limitations. First, the study is limited by the relatively small number of subjects. This is a pilot study with a small sample size, thereby limiting the applicability to populations in Asian countries. However, as different TTCMs are commonly prescribed as skin-whitening agents by traditional Chinese medicine practitioners and are frequently purchased over the counter, our sample size may be sufficient to address issues regarding the frequent adverse effects of TTCMs, particularly, in cosmetics. Second, this study was conducted on healthy volunteers rather than on those with a history of previous sensitisation to TTCMs. Although similar studies had been conducted using subjects with a history of sensitisation, it was not possible in our setting. Third, with regard to plants or herbs with unknown nature or uncertain concentration (many plants or fruits contain crystals and impurities), open application tests, such as patch test or series dilution patch test, may be considered, which may decrease the false positivity of patch test.

Furthermore, to detect low levels of sensitisation, it is suggested to test the individual constituents of a mixture at a higher concentration than those present in the mixture [24]. However, the higher concentration of individual constituent reagents could also result in irritant patch test reaction and deviation from the suggested constituents. In the present study, the positive reaction rate of Radix* Ampelopsis japonica* in the patch test, which was higher than that of BZK, may be owing to the high concentration of the individual reagent (50.0% in petrolatum), which was different from the actual content in BZK (approximately 8.3% of each ingredient in petrolatum). Moreover, patch test using individual constituents was performed at 50% concentration in petrolatum, which was higher than that used in the formula. In the screening series aimed to identify contact sensitivities, a positive reaction to the mixture formula is usually associated with a positive reaction to at least 1 of the individual constituents that rarely gives a false-negative reaction if there is contact sensitivity to the mixture. Thus, it is logical to conclude that reactivity to ≥1 individual constituents contributes to reactivity to the mixture. Nevertheless, in the present study, a positive reaction to the individual constituents might not have necessarily led to reactivity to the mixture, which may be owing to the diluted concentration of the individual constituents in the mixture. This dilution effect may explain the more frequent strong positive reactivity of the subjects to individual constituents than to the mixture.

**Figure 5:**
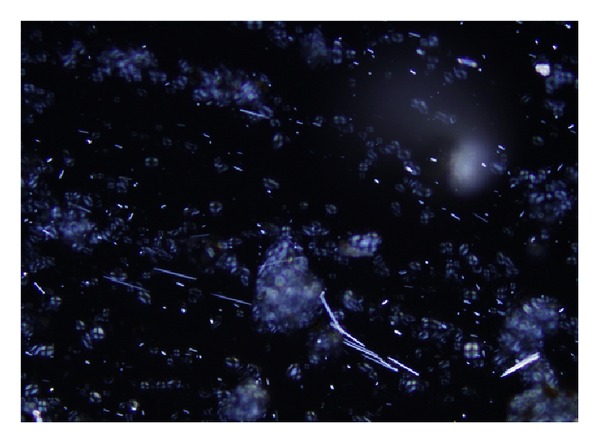
Needle shaped impurities and raphides in Radix* Ampelopsis japonica *preparation under polarizing microscope (400x).

In modern times, herbals are usually used in drugs or cosmetics after extraction. In the present study, we examined the reagents under polarising microscope and found the presence of high quantities of needle-shaped impurities and raphides of Radix* Ampelopsis japonica* rather than BZK (Figure 5). A subsequent patch test experiment with ethanol extraction of Radix* Ampelopsis japonica* (1.0% in petrolatum), which was used to eliminate the impurities and raphides, was conducted 3 months later and was repeated in the 13 subjects who tested positive to Radix* Ampelopsis japonica *or BZK. Interestingly, all the subjects failed to exhibit positive results in the subsequent experiment, suggesting that the needle-shaped impurities and raphides from the herbal powder of Radix* Ampelopsis japonica* might have caused irritant contact dermatitis. Thus, an understanding of these limitations may help to improve future study design. Although it is likely that our findings can be extrapolated to the general population, further studies are needed to confirm our findings, especially with TTCMs after extraction.

## 5. Conclusions

Recently, the potential use of TTCMs in the development of new skincare cosmetics has been emphasised. In the present study, among the constituents of BZK, Radix* Ampelopsis japonica* and Radix* Angelicae dahuricae* appeared to be the sensitisers. Given the vast variety of TTCMs available in the market, ascertaining the safety and therapeutic effects of cosmetic TTCM products and their constituents should be regarded as mandatory. Furthermore, careful use of TTCMs and ample warnings about the risk of contact dermatitis are necessary, particularly, for cosmetic products.

## Supplementary Material

The quality control data of authenticated (appearance, microscopic characteristics and thin-layer chromatography) samples of the individual herbal constituents, complying with specifications given in the Chinese pharmacopoeia, were provided by Sheng Foong Pharmaceutical Co., Ltd (Proof certification according to ISO17025/TAF, Ilan County, Taiwan).

## Figures and Tables

**Table 1:** Results of patch and photopatch tests from 30 subjects.

No.	Gender	Age	Radix *Ampelopsis * *japonica *		Rhizoma *Atractylodis macrocephalae *		Radix *Angelicae dahuricae *		Sclerotium *Poria cocos *		Rhizoma *Bletillae striatae *		*Herba asari *		Bai-Zhi-Kao		Petrolatum	
			PT	PPT	PT	PPT	PT	PPT	PT	PPT	PT	PPT	PT	PPT	PT	PPT	PT	PPT
1	M	38									+	+				+		
2	F	37	+	+												+		
3	F	27																
4	F	64		+			+				+							
5	F	52	+		+										+			
6	F	45	++					+							+			
7	F	40	+															
8	M	37	+					+										
9	F	43	++					+							+			
10	F	38	+															
11	F	36																
12	F	50	+	++												+		
13	M	35	++		+			+							+			
14	F	34	+															
15	F	56	+								+							
16	M	30						+										
17	F	23	++												+			
18	F	23																
19	F	32											+					
20	F	37									+							
21	F	24	+															
22	F	44	+					+										
23	M	29	+		+			+							+			
24	F	22	+															
25	F	21									+							
26	F	25		+				+										
27	F	26																
28	F	30																
29	F	24	+															
30	F	35	+															

Blank: represent negative reaction; PT: patch test; PPT: photo patch test.
