# Supplementary material for: Irritant Contact Dermatitis Risk of Common Topical Traditional Chinese Medicines Used for Skin-Lightening: A Pilot Clinical Trial with 30 Volunteers
Source: Evid Based Complement Alternat Med. 2014 Apr 10;2014:609064. doi: 10.1155/2014/609064 (PMC4003776; doi:10.1155/2014/609064)
Supplement: Supplementary file 1 — The quality control data of authenticated (appearance, microscopic characteristics and thin-layer chromatography) samples of the individual herbal constituents, complying with specifications given in the Chinese pharmacopoeia, were provided by Sheng Foong Pharmaceutical Co., Ltd (Proof certification according to ISO17025/TAF, Ilan County, Taiwan). [file 609064.f1.pdf]

Evidence-Based Complementary and Alternative Medicine

(609064.v2)

Irritant Contact Dermatitis Risk of Common Topical Traditional Chinese Medicines Used for Skin-lightening: A Pilot Clinical Trial with 30 Volunteers

Supplementary Data

Figures. The quality control data of authenticated (appearance, microscopic characteristics and thin-layer chromatography) samples of the individual herbal constituents, complying with specifications given in the Chinese pharmacopoeia, were provided by Sheng Foong Pharmaceutical Co., Ltd (Proof certification according to ISO17025/TAF, Ilan County, Taiwan).

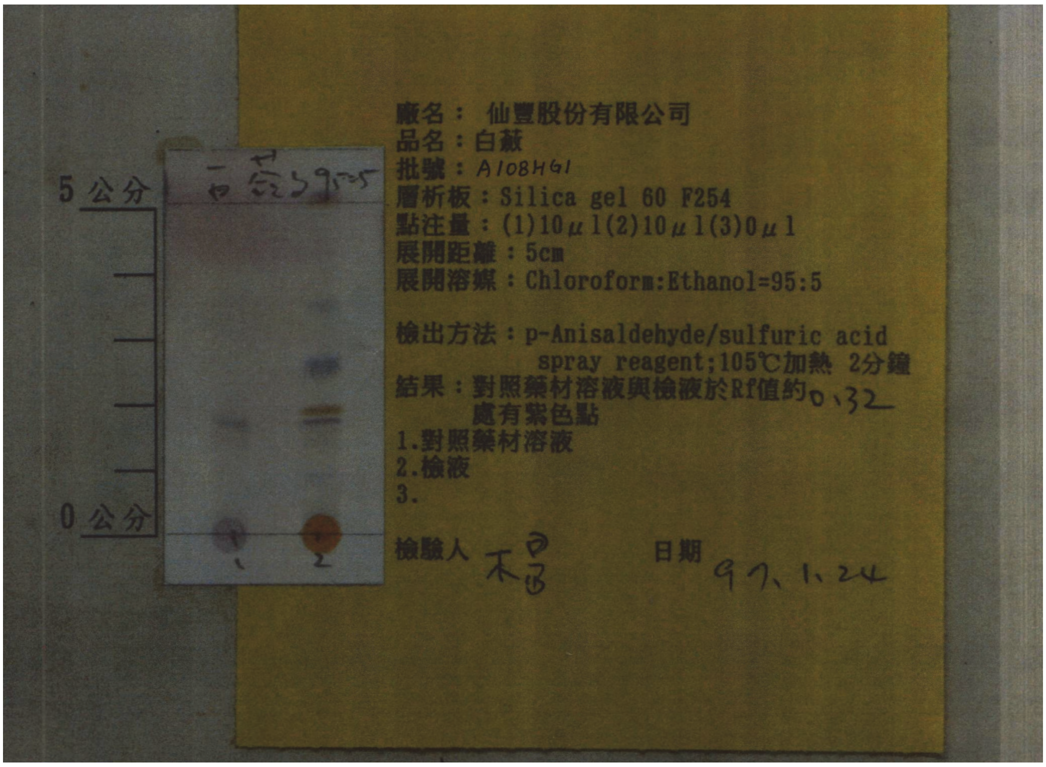

圖3.1 白蘞藥材T.L.C.鑑別檢測

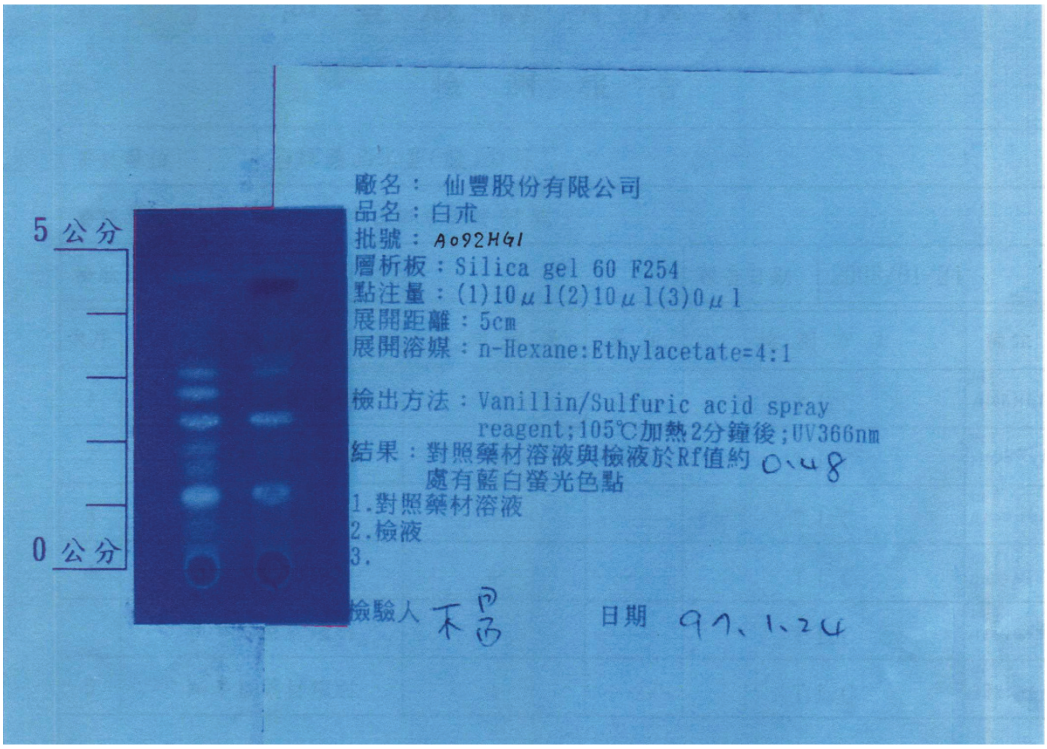

圖3.2 白朮藥材T.L.C.鑑別檢測

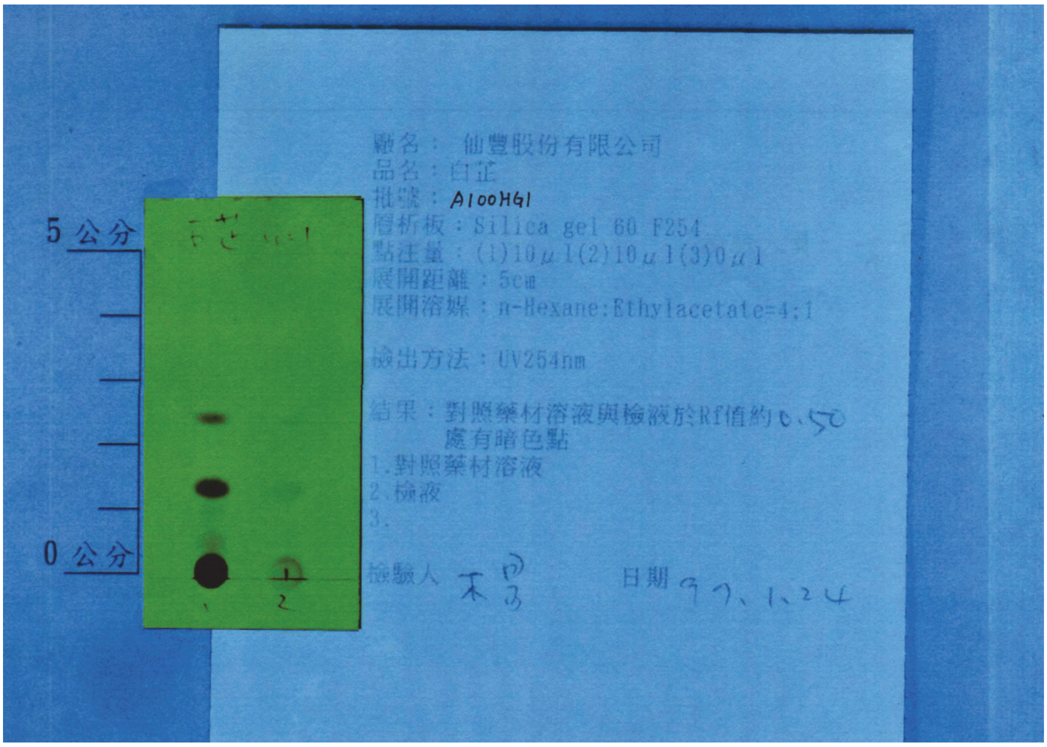

圖3.3 白芷藥材T.L.C.鑑別檢測

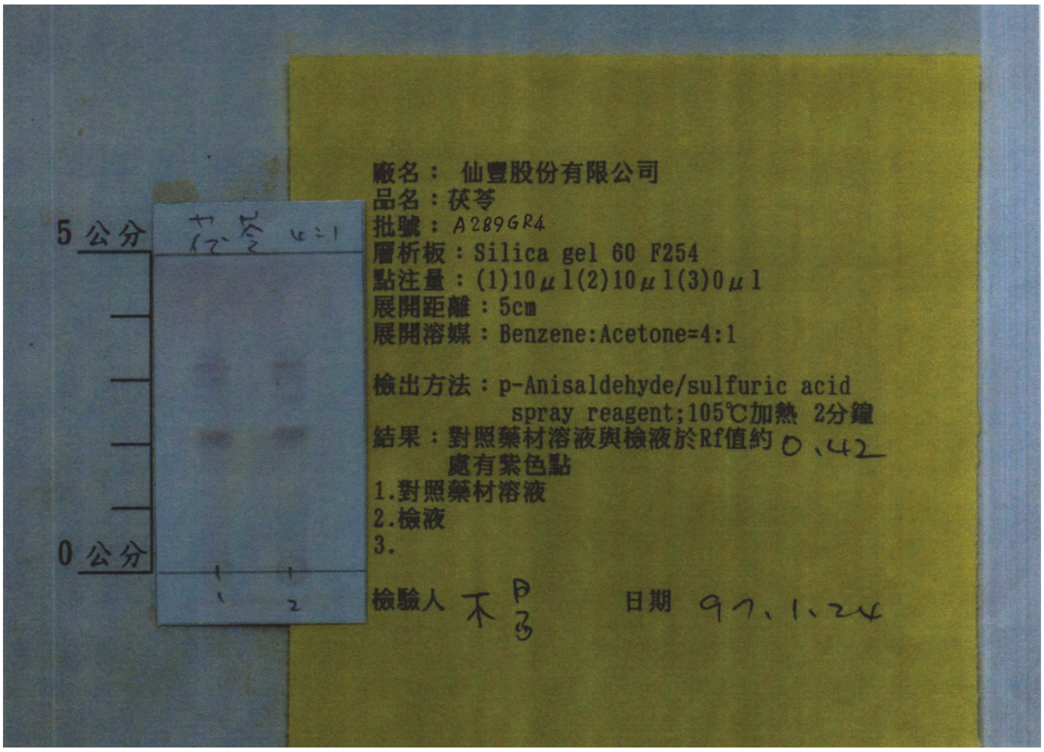

圖3.4 白茯苓藥材T.L.C.鑑別檢測

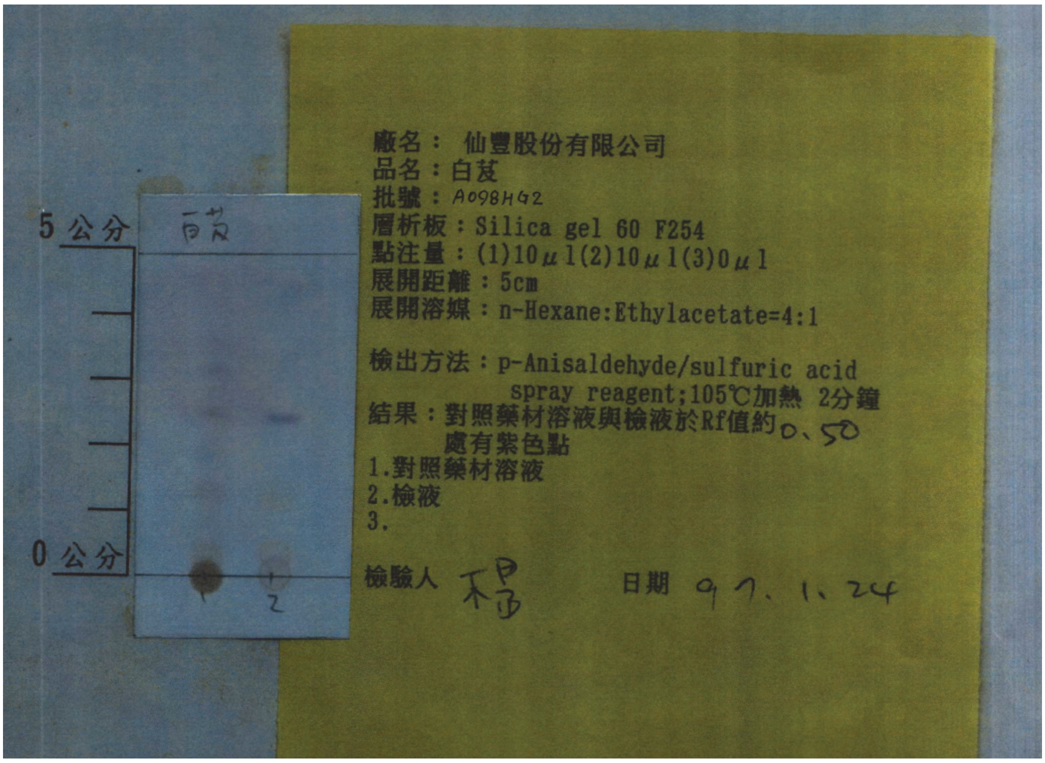

圖3.5 白芨藥材T.L.C.鑑別檢測

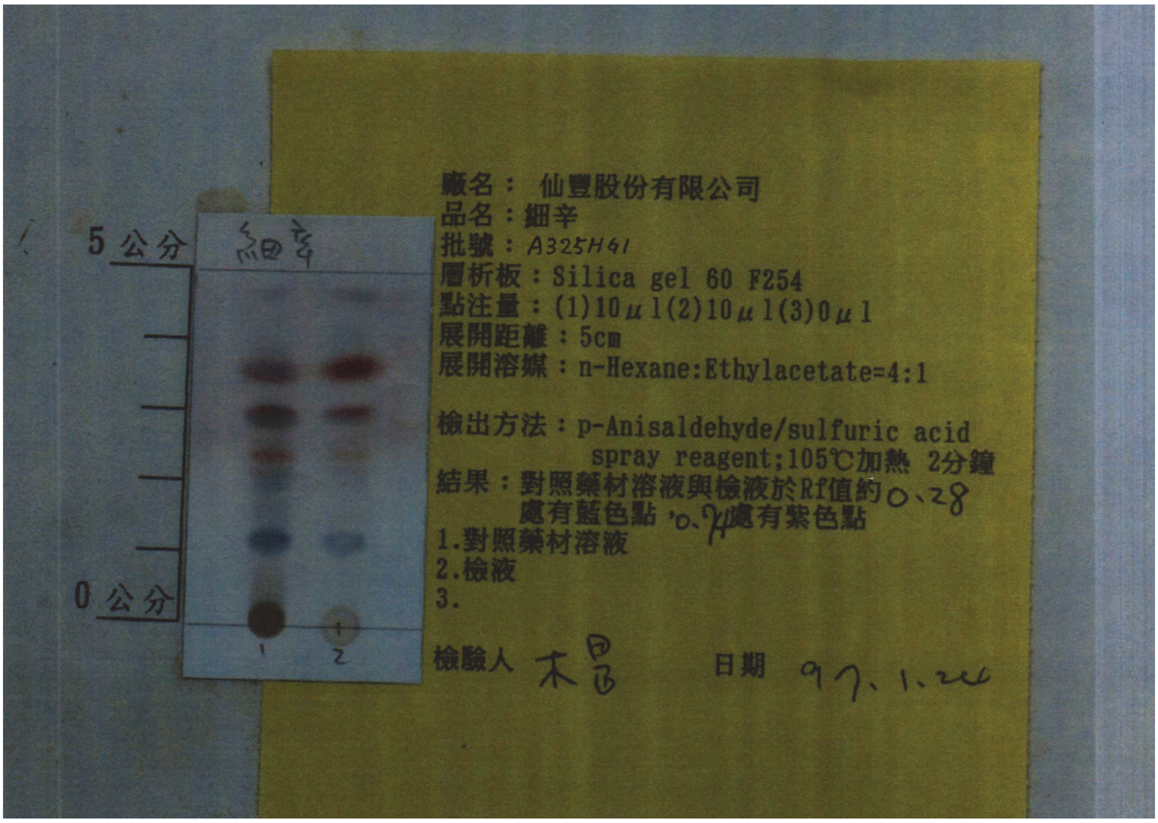

圖3.6 細辛藥材T.L.C.鑑別檢測

仙 豐 股 份 有 限 公 司

檢 測 報 告

委託單位	杏輝藥品工業(股)公司		
委託單位地址	宜蘭縣冬山鄉中山村84號		
檢驗日期	2008/01/24	報告日期	2008/01/24

次序	檢驗項目	檢測結果	單位	檢測方法	備註
1	白蘞片藥材鑑別	+	——	T.L.C	批號: A108H61
2	白朮片藥材鑑別	+	——	T.L.C	批號: A092H41
3	白芷片藥材鑑別	+	——	T.L.C	批號: A100H61
4	白芨片藥材鑑別	+	——	T.L.C	批號: A098H62
5	茯苓片藥材鑑別	+	——	T.L.C	批號: A289624
6	細辛根藥材鑑別	+	——	T.L.C	批號: A325H61

以下空白~~

實驗室主管: [illegible]　　　　檢驗人員: [illegible]

宜蘭縣蘇澳鎮大圳路63巷25號　TEL:(03)9905900　FAX:(03)9905902
No.25, Lane 63, Dah Tzuenn Rd.,Su-Ao Chen, I-Lan County TAIWAN, R.O.C.
